# Median Arcuate Ligament Syndrome: It Is Not Always Gastritis

**DOI:** 10.1177/2324709617728750

**Published:** 2017-09-05

**Authors:** Aneesh Kuruvilla, Ghulam Murtaza, Ayesha Cheema, Hafiz Muhammad Sharjeel Arshad

**Affiliations:** 1 UIC/Advocate Christ Medical Center, Oak Lawn, IL, USA; 2 DHQ Hospital Sargodha, Sargodha, Pakistan

**Keywords:** celiac artery compression syndrome, median arcuate ligament syndrome, abdominal pain

## Abstract

Median arcuate ligament syndrome is a rare disorder that is clinically characterized by the triad of postprandial abdominal pain, weight loss, and often an abdominal bruit due to compression of the celiac artery by the median arcuate ligament. Given the nonspecific symptoms, this is a rare and difficult diagnosis to obtain. We present a patient with nonspecific abdominal pain in whom etiology was ultimately determined to be median arcuate ligament syndrome.

## Introduction

Median arcuate ligament syndrome is a rare disorder that is clinically characterized by the triad of postprandial abdominal pain, weight loss, and often an abdominal bruit due to compression of the celiac artery by the median arcuate ligament. Median arcuate ligament is a fibrous arch that usually passes just superior to the celiac artery near the first lumbar vertebra. In 10% to 24% of the general population, the ligament crosses anterior to the celiac artery, and a few of these patients may have hemodynamically significant stenosis causing abdominal pain.^1^ Given the nonspecific symptoms, this is a rare and difficult diagnosis to obtain. We present the case of a patient with nonspecific abdominal pain in whom etiology was ultimately determined to be median arcuate ligament syndrome.

## Case Presentation

A 38-year-old woman with no known past medical history presented to the emergency room with diffuse, cramping, nonradiating abdominal pain episodes lasting for 30 to 60 minutes. The patient also had associated symptoms of nausea and bilious vomiting, which temporarily relieved the pain. These episodes had no known precipitating factor and were not associated with food or exercise. She had 2 similar episodes in the past and was admitted to hospital 4 months ago. Imaging and labs at that time were normal. She denied any significant alcohol, tobacco, or drug use. She also denied any significant family medical history. Her physical examination revealed mild epigastric tenderness to palpation but no other abnormalities.

**Figure 1.**
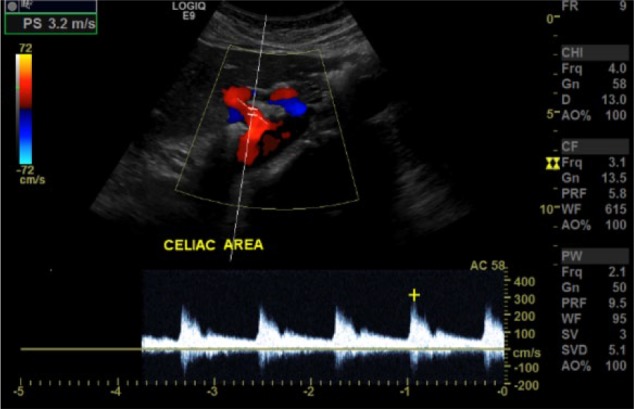
Mesenteric ultrasound of celiac artery with velocity of 320 cm/s.

**Figure 2.**
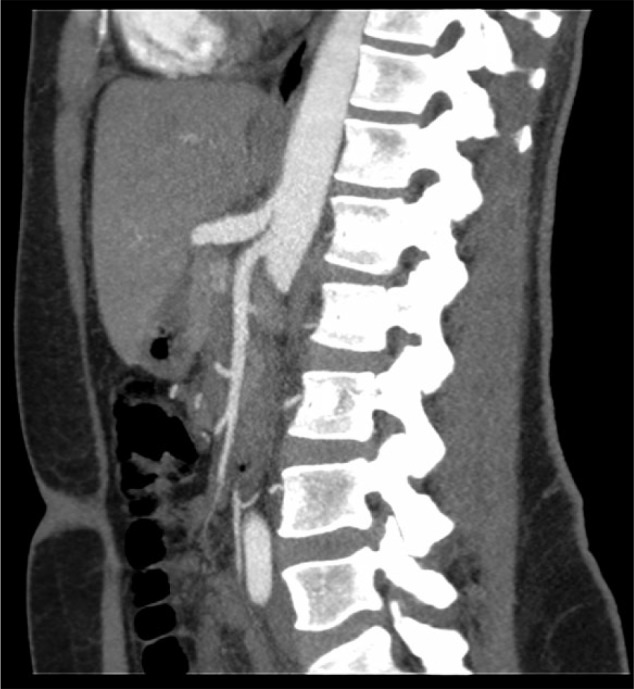
Sagittal view of computer tomography angiography showing focal stenosis at proximal part of the celiac axis.

Complete blood counts, liver function tests, serum lipase, and urinalysis were all within normal limits. Esophagogastroduodenoscopy showed mild antral gastritis. However, pantoprazole failed to relieve severe pain episodes. A mesenteric ultrasound was ordered, which demonstrated increased celiac axis flow velocities with a peak systolic velocity of 320 cm/s (Figure 1). These findings were suggestive of significant stenosis greater than 70%. A computed tomography (CT) scan of abdomen with contrast showed superior compression of celiac axis with focal narrowing at origin and poststenotic dilation (Figure 2). These findings confirmed the diagnosis of median arcuate ligament syndrome. The patient was referred to surgery for a laparoscopic release of median arcuate ligament.

## Discussion

Median arcuate ligament syndrome (MALS) is a very rare and difficult diagnosis to make due to its nonspecific symptoms and presentation. MALS is also referred to as celiac artery compression syndrome or Dunbar syndrome. MALS was first described by Harolja in 1963 in a patient who presented with abdominal pain that was attributed to mesenteric ischemia caused by compression of the celiac artery by a low-lying median arcuate ligament.^1^

Even though the etiology of MALS is not well understood, there are 2 main theories, mainly mesenteric ischemia and nerve dysfunction.^2,3^ One of the proposed theory is compression of the celiac artery by a median arcuate ligament that causes mesenteric ischemia, which leads to classical symptoms of MALS.^2^ However, It is not well known how celiac artery compression alone can cause mesenteric ischemia since generally there is extensive collateral blood supply to the mesenterium from other blood vessels.^4^ Another proposed mechanism for MALS is due to celiac plexus nerve dysfunction.^3^

Although in CT analysis compression of celiac artery is observed in up to 25% of patients, yet clinically symptomatically MALS is very rare.^1^ Patients are generally young woman between 30 and 50 years of age who have had multiple workups for abdominal pain. Abdominal pain is usually located in the epigastric area and worse after food. Even though there are no unique physical exam findings to MALS, an epigastric bruit may be observed in up to 35% of the symptomatic patients.^3^ In our patient, her abdominal pain was diffuse and she was tender to palpation on the epigastric area. Abdominal bruit was absent.

Since symptoms of MALS are nonspecific and overlap with other causes of abdominal pain, it is a diagnosis of exclusion. There are different imaging modalities that are used to diagnose MALS, such as mesenteric ultrasound, computed tomography angiography (CTA), magnetic resonance angiogram (MRA), and conventional angiography. Although conventional angiography is considered that gold standard for the diagnosis of MALS, angiography has largely been replaced by CTA and MRA.^5^ Sagittal view CTA or MRA will be able to detect focal narrowing of the proximal celiac axis.^5^ In our patient, CTA demonstrated focal narrowing of the celiac axis at the origin with a poststenotic dilation along with some degree of superior compression of the celiac axis suggestive of MALS.

Mesenteric ultrasound is another modality that is used to diagnose MALS. Performed during deep expiration, duplex ultrasound shows increased blood flow velocity across the compressed area of the celiac artery supports the presence of constriction.^6^ Combination of maximum expiratory peak velocity of >350 cm/s and deflection angle greater than 50° seems to be most reliable indicator for MALS on ultrasound.^6,7^

Surgical decompression of the median arcuate ligament is the treatment of choice for symptomatic MALS patients. There are different modalities of surgery. Open surgery with decompression of the celiac artery and celiac plexus by division of the median arcuate ligament fibers is the most common treatment. However, laparoscopic and robotic-assisted laparoscopic approaches have also been utilized successfully and are recently getting popular.^8^ Typically, pain relief is immediate after the surgery. However, outcomes following surgical decompression are varied.^9^ There are multiple case reports with abdominal pain that did not resolve immediately after surgery.^3,10^ Since postsurgical pain can mimic presurgical symptoms, it may take up to 1 month for complete resolution of symptoms.^8^ Therefore, patients should be closely observed after surgery.

## Conclusion

This case illustrates that MALS is an often missed diagnosis due to its nonspecific symptoms. Although MALS is a diagnosis of exclusion, when evaluating patients, especially young females, who present with abdominal pain of unclear etiology, MALS should be considered. MALS should be evaluated with a mesenteric ultrasound to assess celiac artery velocities. Patients with elevated velocities on mesenteric ultrasound should undergo confirmatory test with CTA or angiography. Patients who are diagnosed with MALS should be referred to surgery for decompression of the median arcuate ligament.

## References

[1] Duffy AJ Panait L Eisenberg D Bell RL Roberts KE Sumpio B Management of median arcuate ligament syndrome: a new paradigm. Ann Vasc Surg. 2009;23:778-784. 19128929 10.1016/j.avsg.2008.11.005

[2] Reuter SR Bernstein EF The anatomic basis for respiratory variation in median arcuate ligament compression of the celiac artery. Surgery. 1973;73:381-385. 4687796

[3] Jimenez JC Harlander-Locke M Dutson EP Open and laparoscopic treatment of median arcuate ligament syndrome. J Vasc Surg. 2012;56:869-873. doi 10.1016/j.jvs.2012.04.057 http://www.sciencedirect.com/science/article/pii/S0741521412010440. Accessed August 22, 2017. 22743019

[4] de Lara FV Higgins C Hernandez-Vila EA Median arcuate ligament syndrome confirmed with the use of intravascular ultrasound. Tex Heart Inst J. 2014;41:57-60. doi:10.14503/THIJ-12-2495. 24512402 PMC3967474

[5] Lainez RA Richardson WS Median arcuate ligament syndrome: a case report. Ochsner J. 2013;13:561-564. 24358009 PMC3865839

[6] Tembey RA Bajaj AS Wagle PK Ansari AS Real-time ultrasound: key factor in identifying celiac artery compression syndrome. Indian J Radiol Imaging. 2015;25:202-205. doi:10.4103/0971-3026.155882. 25969647 PMC4419433

[7] Gruber H Loizides A Peer S Gruber I Ultrasound of the median arcuate ligament syndrome: a new approach to diagnosis. Med Ultrason. 2012;14:5-9. 22396932

[8] Roseborough GS Laparoscopic management of celiac artery compression syndrome. J Vasc Surg. 2009;50:124-133. doi:10.1016/j.jvs.2008.12.078 http://www.sciencedirect.com/science/article/pii/S0741521409000068. Accessed August 22, 2017. 19563960

[9] Columbo JA Trus T Nolan B et al Contemporary management of median arcuate ligament syndrome provides early symptom improvement. J Vasc Surg. 2015;62:151-156. doi:10.1016/j.jvs.2015.01.050 http://www.sciencedirect.com/science/article/pii/S0741521415001603. Accessed August 22, 2017. 25758451 PMC5292272

[10] Reilly LM Ammar AD Stoney RJ Ehrenfeld WK Late results following operative repair for celiac artery compression syndrome. J Vasc Surg. 1985;2:79-91. doi:10.1016/0741-5214(85)90177-6 http://www.sciencedirect.com/science/article/pii/0741521485901776. Accessed August 22, 2017. 3965762

